# The effect of bioactive membrane in alveolar ridge preservation: A double‐blind randomized clinical trial

**DOI:** 10.1002/jper.70094

**Published:** 2026-02-26

**Authors:** Magdalena Orlowska, Muhammad H. A. Saleh, Ann Decker, Junying Li, Alice Ou, Nataly Zambrana, Paolo Nava, Fernanda A. da Costa Deller, Hom‐Lay Wang

**Affiliations:** ^1^ Department of Periodontics and Oral Medicine University of Michigan School of Dentistry Ann Arbor Michigan USA; ^2^ State University of Maringa Maringa Parana Brazil

**Keywords:** bone regeneration, dental implants, heterografts, tooth extraction

## Abstract

**Background:**

This randomized controlled trial aims to evaluate alveolar ridge preservation (ARP) with or without a biologically active amnion–chorion membrane (BACM).

**Methods:**

Thirty patients requiring tooth extraction in the esthetic zone were randomly assigned to 2 groups. Both groups underwent tooth extraction and collagenated bovine bone grafting, while the test sites received an additional BACM. Cone‐beam computed tomography (CBCT) and intraoral scans (IOS) were performed before extraction and at 4 months post‐extraction. Patient‐reported outcomes (PROs) were assessed. The primary endpoint was the change in vertical and horizontal hard and soft tissue dimensions as measured by CBCT and clinically.

**Results:**

All patients completed implant placement. CBCT measurements at 2 mm demonstrated that control and test groups exhibited reductions in horizontal ridge width with median decreases of 1.19 mm and 2.41 mm, respectively, with no significant differences (*p* = 0.126). For vertical height changes, the control and test groups experienced mean reductions of 0.99 ± 1.08 mm and 1.33 ± 0.73 mm, respectively, with no significant differences (*p* = 0.250). IOS analyses showed that mean soft tissue thickness at the alveolar crest was 2.67 ± 0.699 mm (*p* = 0.370) in the control versus 2.13 ± 0.807 mm (*p* = 0.846) in the test group, with no significant differences (*p* = 0.325).

**Conclusion:**

The addition of a BACM did not improve the mean horizontal or vertical bone dimentional changes or have a measurable effect on the soft tissue outcome during ARP.

## Plain language summary

In this clinical study, adults who needed a front tooth or premolar extracted and later replaced with an implant had the socket filled with the same bone graft plug but were randomly assigned to either to receive a standard collagen plug (control) or the same collagen plug covered with a biologically active amnion–chorion membrane (test). Thirty patients completed treatment, and the researchers used 3D X‐rays (cone‐beam computed tomography [CBCT]) and clinical measurements taken at extraction and about 4 months later to track how much bone and gum tissue was lost. They also measured how well the sites healed, how much pain patients felt, whether extra grafting was needed, and how satisfied patients were. Both groups showed some normal shrinkage of the ridge over time, but the amount of horizontal and vertical bone loss and soft tissue thickness was very similar between the 2 treatments, and differences were not statistically significant. Wound healing scores and pain levels improved over time in both groups, with the BACM group looking a bit better at some time points, but again without meaningful statistical differences. The takeaway is that adding an amnion–chorion membrane on top of a xenograft and collagen plug in these uncomplicated extraction sites did not produce clearly superior hard‐ or soft‐tissue outcomes or patient‐reported experience compared with the simpler collagen plug approach.

## 1 INTRODUCTION

Multiple factors influence the selection of biomaterials at the time of alveolar ridge preservation (ARP), including the anatomical features of the socket, the size and shape of the defect (if present), predicted timing of implant placement (IP), and patient preferences such as religious, ethnic, or cultural considerations, and the surgeon's preference.^1^, ^2^ Each graft material offers specific advantages and disadvantages regarding resorption rate, osteoconductivity, and cost.^3^, ^4^

Xenografts composed of deproteinized bovine bone mineral (DBBM) are renowned for their biocompatibility.^5^ Formulations combining bovine particulate xenograft with type I collagen have demonstrated significant advantages in terms of healing time, dimensional stability, and bone regeneration for ARP.^4^, ^6^ Histologically, these collagenated xenografts support osteoblast activity, vascularization, and tissue regeneration.^7^, ^8^

Another variable to consider in ARP is the use of a barrier membrane.^9^ Using exposed barrier membranes in ARP is well documented and shows high percentages of vital bone and favorable ridge dimensional changes.^10^, ^11^, ^12^ Amnion–chorion membrane (ACM) is an allograft derived from human placental tissue after a full‐term, healthy delivery, typically obtained after a cesarean section. The membranes are typically processed to remove cells (e.g., by decellularization) while retaining the extracellular matrix, which is rich in collagen, growth factors, and cytokines.^13^ This matrix is what provides therapeutic benefits in various medical applications, such as promoting healing and tissue regeneration.^14^, ^15^ The pluripotency properties of ACM cells allow them to differentiate into numerous cell types, including osteoblasts,^16^ and are proposed to reduce bacterial contamination and decrease the risk of postsurgical inflammation.^17^, ^18^, ^19^ Lastly, the amnion layer of ACM contains functional cytokines that are important for cell signaling, such as fibroblast growth factor (FGF), platelet‐derived growth factor (PDGF), keratinocyte growth factor (KGF), basic FGF (bFGF), and transforming growth factor beta (TGFß) ^20^; all of which have been proposed to improve tissue healing and overall quality.^21^, ^22^

The favorable biological properties of ACM support their use as barrier membranes for ARP. A randomized split‐mouth trial comparing ACM with d‐PTFE membranes found similar overall ridge preservation (RP) outcomes but demonstrated that ACM led to significantly greater bone volume density and osteoid formation at 3 months on histomorphometric analysis.^23^

The Implant Dentistry Core Outcome Set and Measurement (ID‐COSM) international consensus report has recommended specialized core outcome domains for bone augmentation trials (BA‐COSM).^24^ Pertinent to ARP, the consensus established that certain outcomes are mandatory, such as assessment of surgical complications and adverse events, dimensional bone changes, and the ability to place implants in a prosthetically guided position. Other important but optional domains included histology.^24^

Hence, the primary aim of this study was to evaluate the superiority of using a biologically active amnion‐chorion membrane (BACM) in the change in vertical and horizontal hard tissue dimensions after ARP as measured by cone‐beam computed tomography (CBCT) and clinically. Secondary endpoints included vertical and horizontal soft tissue measurements, wound healing assessments, patient‐reported outcome measures (PROMs), bone density, horizontal ridge dimensional changes after IP as measured clinically, the need for additional grafting, and overall patient satisfaction.

## 2 MATERIALS AND METHODS

### 2.1 Trial design

This clinical trial was parallel, randomized, controlled, and double‐blinded (subject and assessor blinded). Measurements (clinical and radiological) throughout the study were performed by blinded assessors (M.O., N.Z., and P.N., respectively). Recruited individuals underwent tooth extraction and xenogeneic bone graft placement, followed by a collagen plug (CM) only (Control) or BACM on top of the CM (test). The study was conducted from August 4th, 2022, to June 28th, 2024.

**FIGURE 1:**
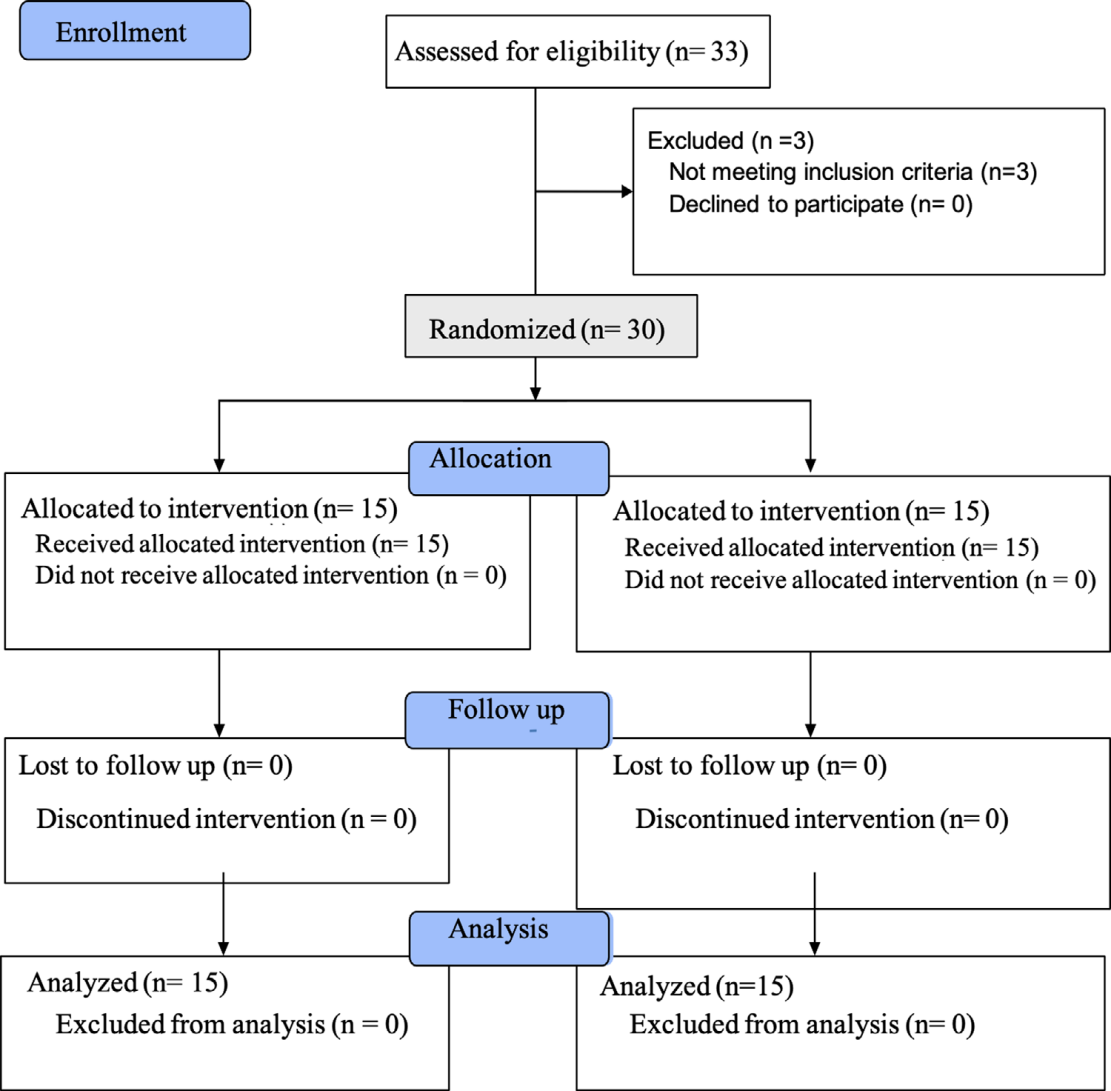
Flowchart for clinical trial enrollment. CONSORT 2010 flow diagram.

The study was performed according to the CONSORT statement (http://www.consort‐statement.org/) (Figure 1) following the CONSORT guidelines^25^ and to the principles outlined in the Declaration of Helsinki on experimentation involving human subjects, as revised in 2000. The Institutional Review Board (IRB) for the Medical Sciences at the University of Michigan, Ann Arbor, MI, reviewed and approved this study before participant enrollment began (IRBMed HUM00208140). This study was also registered on ClinicalTrials.gov (Registration# NCT05592158) prior to commencement. The flowchart of time points for data collection is included in Supplemental Figure S1 and Appendix.

### 2.2 Participants

From the patient population presenting at the University of Michigan School of Dentistry requiring extraction and IP, subjects were screened by the same researcher (M.O.) according to preset eligibility criteria.

*Inclusion criteria*

- Healthy adults (≥18 years old, <80 years), presenting with good oral hygiene (plaque index of <10%),^26^ and willing to comply with all study visits.
- Patients must require tooth extraction (for incisors, canines, and premolars) due to caries, tooth fracture, or teeth deemed un‐restorable.
- The extraction socket should be free of infection, <5 mm of buccal bone dehiscence (class 1 and 2A sockets (Chu et al., 2015).^27^
- Patients interested in IP following tooth extraction.

*Exclusion criteria*

- Heavy smokers (>10 cigarettes/day).
- Active periodontitis
- Teeth that required extraction due to periodontitis.
- Known allergies or hypersensitivities to the study‐related medications such as chlorhexidine.
- Severe hepatic or renal dysfunction.
- Uncontrolled diabetes (HbA1c > 8.5).
- Patients on chronic pain medications.
- Active cancer treatment, including chemotherapy or radiation therapy.
- Use of medications known to hinder bone healing, such as bisphosphonates and prolonged anti‐inflammatory drugs.
- Presence of metabolic bone diseases impacting bone healing, like osteoporosis (as reported by the participant).
- Pregnancy or breastfeeding (as reported by the participant).
- Metabolic bone diseases that affect bone healing (osteoporosis self‐reported).

Prior to any intervention, the potential risks and benefits of the procedure were reviewed with the participants, and written consent was obtained.

#### 2.2.1 Groups: Test and control/randomization

Subjects were randomly assigned to one of the treatment groups using a concealed draw method, with each card corresponding to a specific group. Randomization was completed after tooth extraction. Surgeons were blinded to group assignments until the surgical intervention (placing xenograft and collagen plugs with or without a membrane) commenced. Participants and assessors remained blinded until the end of the study. If desired, participants were informed of their group allocation at the study's conclusion.

A bovine‐origin xenograft bone substitute material (MinerOss X Plug composed of 80% bovine‐derived cancellous particulate xenograft and 20% bovine type I collagen) was used in both groups.

CP control group: Atraumatic extraction, bone grafting using a xenogeneic plug in the extraction socket (MinerOss X Plug, Biohorizons, AL, USA), CP (Bioplug, Biohorizons, AL, USA) and suturing polytetrafluoroethylene (PTFE) over the socket.

BACM test group: Atraumatic extraction, bone grafting using a xenogenic plug in the extraction socket (MinerOss X Plug, Biohorizons, AL, USA), CP (Bioplug, Biohorizons, AL, USA), with the additional usage of BACM (Mem‐Lok Amnio, Biohorizons, AL, USA) that covered the CP and suturing (PTFE) over the socket.

Both groups received a MinerOss X plug covered with a collagen bioplug. In the BACM (test) group, an additional amnion‐chorion membrane was placed over the MinerOss X plug and collagen bioplug, whereas in the control group, no membrane was applied.

Our hypothesis is that there is a statistically significant clinical and radiographic outcome difference based on the analysis of the 2 groups.

### 2.3 Interventions

#### 2.3.1 Ridge preservation (Please refer to the Appendix)

**FIGURE 2:**
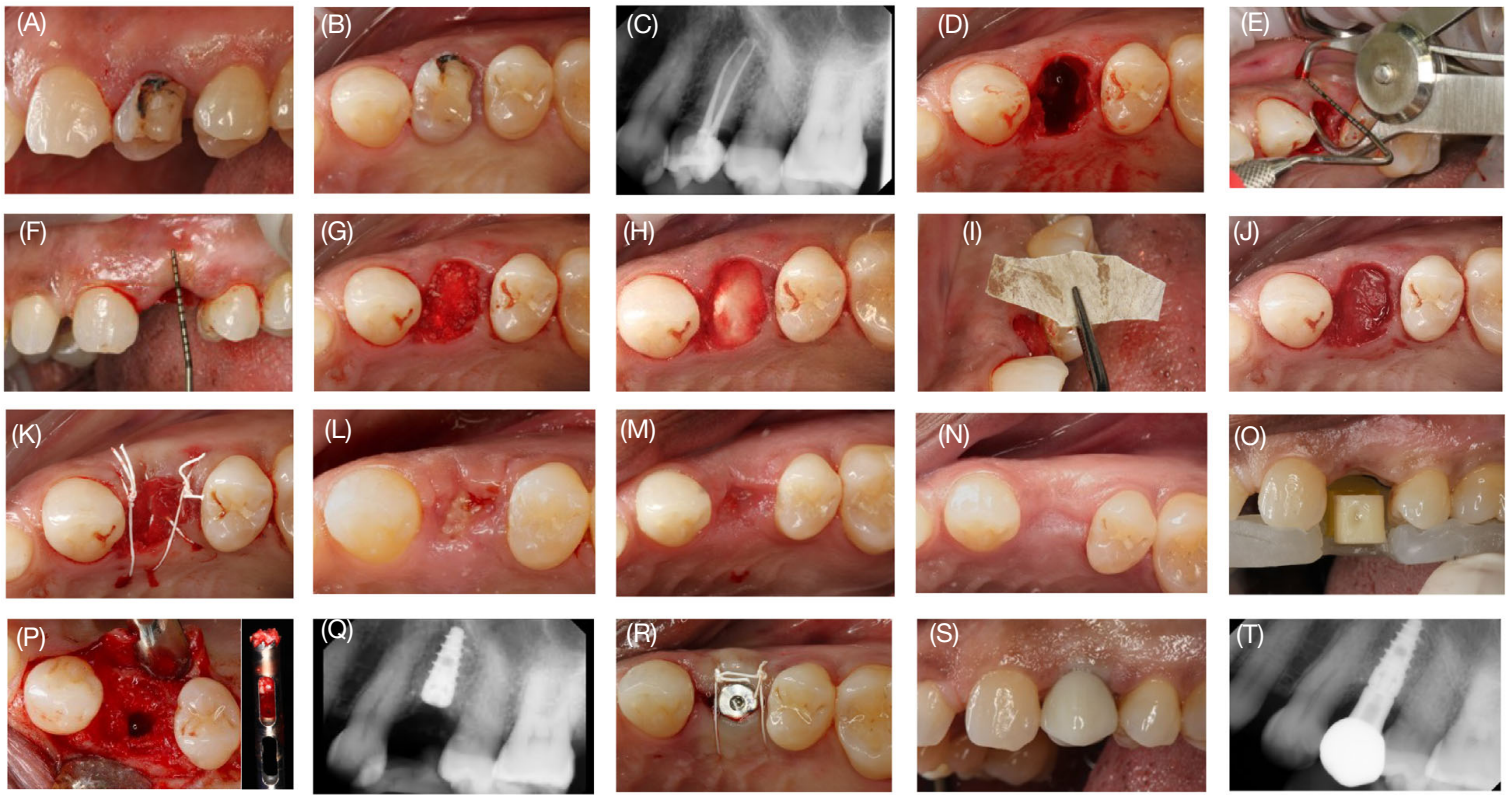
Multipanel illustrating the sequence of treatment in the test group. (A) Baseline, lateral view. (B) Baseline, occlusal view. (C) Baseline standardized PA X‐ray. (D) Minimally traumatic tooth extraction. (E, F) Measurements after extraction using caliper and periodontal probe. (G) Socket filled with xenogenic bone graft. (H) Socket sealed with collagen plug. (I, J) Socket sealed with a BACM above the collagen plug (only in test group). (K) 3.0 PTFE Crisscross sutures. (L) Postoperative follow‐up at 2 weeks. (M) Postoperative follow‐up at 4 weeks. (N) Postoperative follow‐up at 12 weeks. (O) Surgical guide try in. (P) Full‐thickness periosteal flap and bone core biopsy sample obtained prior to implant placement. (Q) Two‐stage implant placement approach. Standardized PA X‐ray after implant placement. (R) Healing abutment placement 4 months after implant placement. (S) Final crown delivery. (T) Standardized PA X‐ray after crown delivery.

A standardized initial radiograph of the surgical area (Figure 2A and 2B) was taken using a paralleling technique (XCP; Rinn Corp., Elgin, IL, USA; Figure 2C) to ensure accurate imaging and minimize distortion. To standardize the X‐ray procedure, impression material was applied to the X‐ray bite block, allowing the patient to bite down in the same way each time. This ensured that the X‐ray ring and sensor remained in the exact same position for each imaging session, providing reproducible imaging. Plaque^28^ and gingival index^29^ scores were recorded at the start and during each subsequent visit. An initial CBCT scan was performed to measure each patient's initial ridge dimensions. Patients received presurgical instructions regarding the following visits.

Two experienced surgeons (H.L.W. and M.S.) performed all surgical procedures and clinical measurements and were calibrated prior to and throughout the study to ensure measurement of the same parameters with high accuracy and reliability (Supplemental Table S1).

Once minimally traumatic extraction was performed (Figure 2D), soft tissue thickness, measured at 2 and 4 mm apical to the ridge crest, was assessed using an endodontic K‐flex file (#30) with a rubber stopper. The distance indicated by the rubber stopper was then measured using an endodontic finger ruler (Hu‐Friedy, Chicago, IL, USA) and rounded to the nearest 0.25 mm. Buccal and lingual bone thickness and ridge width were measured using a caliper 2 and 4 mm below the crest after subtracting the previously measured tissue thickness (Figure 2E and Supplemental Figure S2). Keratinized tissue width was measured on the buccal site using a periodontal probe (UNC Probe; Hu‐Friedy; Figure 2F, Supplemental Figure S2D–F).

Bone grafting was performed using a xenogeneic plug in the alveolus (MinerOss X Plug, Biohorizons, AL, USA) and condensed lightly into the socket (Figure 2G). A collagen plug (Bioplug, Biohorizons, AL, USA) was placed to contain the bone graft (Figure 2H). At this stage, the surgeon was informed if the patient was in the BACM test group, the collagen plug was covered with a BACM (Mem‐Lok Amnio, Biohorizons, AL, USA; Figure 2I, J). A crisscross suture was placed over both sites, but no attempt was made to gain primary closure (Figure 2K).

Patients received verbal postoperative instructions and were prescribed systemic antibiotics. Amoxicillin 500 mg every 8 hours for 7 days, if allergic, azithromycin 250 mg 2 tablets the first day, then 1 tablet/day for the remaining 4 days, and ibuprofen 600 mg, every 6 hours for 3 to 5 days. Patients were also asked to complete a pain survey for 1 week (Appendix). Two‐, 4‐, and 12‐week follow‐ups were scheduled. (Figure 2I, M, N).

#### 2.3.2 IP procedures (please refer to the Appendix, supplemental Table S8, supplemental Table S9, supplemental Figure S8)

Reentry procedures were performed 4 months (± 2 weeks) after ARP to place a dental implant. Prior to the surgical intervention, a new standardized radiograph, digital impression, CBCT scan, and surgical guides were prepared (Figure 2O, supplemental Figure S8).

### 2.4 Outcomes

For outcome measures, the primary endpoint of the present study was the change in vertical and horizontal hard tissue dimensions after ARP as measured by CBCT and clinically. Secondary endpoints included vertical and horizontal soft tissue measurements, wound healing assessments, PROs, bone density, horizontal ridge dimensional changes after IP as measured clinically, the need for additional grafting, and overall patient satisfaction.^30^ Supplemental Table S1 shows the same outcomes in chronological order as measured throughout the clinical trial.

#### 2.4.1 Horizontal and vertical hard and soft tissue outcomes after ARP

##### 2.4.1.1 CBCT measurements (Figure 3f–h). (For details, please refer to the appendix)

**FIGURE 3:**
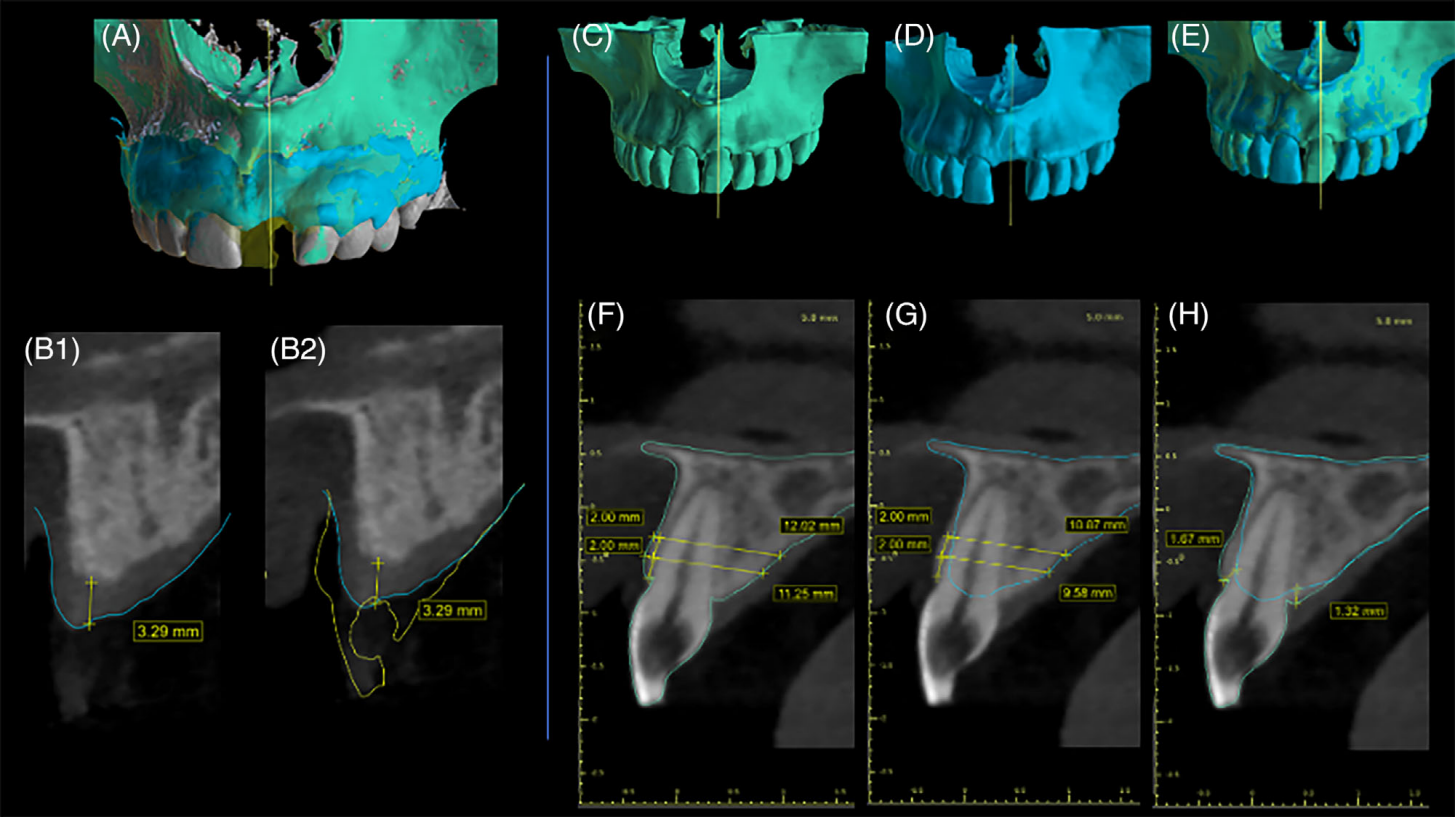
(A) Mid‐sagittal reference slice over 3D CBCT reconstructions superimposed with intraoral scans. (B) CBCT and intraoral scans with soft tissue mid‐crestal thickness. (B1) CBCT and intraoral scan 4 months after extraction; (B2) additional superimposition of baseline intraoral scan, horizontal and vertical differences are seen between intraoral scan. (C–E) Segmented CBCT as bone surfaces. (C) Left, Baseline bone surface. (D) Four months after extraction bone surface. (E) Aligned bone surfaces. (F–H) Bone surfaces outlines superimposed to the baseline CBCT mid‐sagittal slice. Vertical measurements of 2 mm from the buccal crest (2 and 4 mm) are shown for horizontal measurements references. (F) Baseline CBCT and baseline bone surface superimposed, horizontal measurements made following 2 and 4 mm references. (G) Baseline CBCT and 4 months after extraction bone surface superimposed with horizontal measurements. (H) Baseline CBCT, baseline and 4 months after extraction bone surfaces superimposed. Buccal and palatal vertical measurements of bone surface differences.

All patients underwent 2 CBCT scans at 2 time points: before extraction and 4 months post‐extraction, using a 3D Accuitomo machine (J. Morita Corp, Japan). The baseline CBCT assessed the tooth position, horizontal bone thickness, dehiscence, and surrounding anatomical structures. The post‐extraction CBCT evaluated the site for IP. To assess horizontal and vertical bone changes, automatic segmentation of the full arch and point‐based alignment using the implant planning software Blueskyplan v.4.12.13 (Blueskybio, USA) was performed and blindly assessed by the same investigator (N.Z.). Baseline and 4 months after extraction CBCT were automatically segmented as bone surfaces and exported as standard triangulation language (STL) files. A mid‐sagittal slice representative of the extracted tooth was chosen to evaluate changes of horizontal and vertical bone remodeling. The baseline CBCT and bone surfaces were aligned so outlines of baseline and 4 months after extraction are shown over the CBCT slice mid‐sagittal slice and all measurements were made on the same slice to avoid angulations measurements. The radiographic parameters measured were as follows:

- Horizontal ridge width (mm) at 2 mm apical to crest (CBCTCRW2) and 4 mm apical to crest (CBCTCRW4) at baseline and 4 months after extraction
- Vertical buccal/lingual cortical plate measurements (mm) assessed at 1 time point (4 months after extraction) (Figure 3f–h).

##### 2.4.1.2 Clinical Measurements

The clinical parameters were measured at 2 time points: after extraction and at the re‐entry

Surgery (Table 1) as follows:

- Keratinized tissue (KT) width at the buccal site
- Gingival thickness (GT) at 2 (GT2) and 4 mm (GT4) below the ridge crest at the buccal surface
- Horizontal ridge width at 2 and 4 mm below the crest.

**TABLE 1:** Demographic and implant site parameters and its influence on clinical and radiographic parameters.

Parameter	Control group	Test group	Total	*p*‐value
**Demographic homogeneity**				
**No. of subjects (%)**	15 (50%)	15 (50%)	30 (100%)	
**Sex**				1.000 (Chi2)
Female (%)	6 (40%)	6 (40%)	12 (40%)	
Male (%)	9 (60%)	9 (60%)	18 (60%)	
**Age (years)**				
Median (range)	60 (33–77)	65 (24–75)	62 (24–77)	0.305 (MW)
Mean ± SD	55.8 ± 14.5	60.7 ± 13.9	58.2 ± 14.2	
**Arch (number, %)**				1.000 (Fisher)
Maxilla	12 (80%)	12 (80%)	24 (80%)	
Mandible	3 (20%)	3 (20%)	6 (20%)	
**Implant site homogeneity**				
**Buccal site KG baseline (mm)**				
Median (range)	5 (1–6)	4 (3–7)	5 (1–7)	0.775 (MW)
Mean ± SD	4.30 ± 1.72	4.60 ± 1.47	4.45 ± 1.58	
**Buccal BT 2 mm baseline (mm)**				
Median (range)	0.5 (0–1.5)	0.5 (0–1.6)	0.5 (0–1.6)	0.486 (MW)
Mean ± SD	0.51 ± 0.54	0.65 ± 0.5	0.58 ± 0.51	
**Buccal BT 4 mm baseline (mm)**				
Median (range)	1.15 (0.2–1.5)	1 (0.3–4)	1 (0.2–4)	0.847 (MW)
Mean ± SD	1.05 ± 0.4	1.28 ± 0.93	1.17 ± 0.72	
**Lingual BT 2 mm baseline (mm)**				
Median (range)	0.8 (0–2.5)	1.5 (0–4)	1.10 (0–4)	**0.026** ^*^ **(MW)**
Mean ± SD	0.87 ± 0.73	1.64 ± 1.05	1.26 ± 0.97	
**Lingual BT 4 mm baseline (mm)**				
Median (range)	1.9 (0.4–4.5)	2.6 (0.2–5)	2.3 (0.2–5)	0.201 (MW)
Mean ± SD	2.17 ± 1.04	2.63 ± 1.28	2.38 ± 1.16	
**Buccal STT 2 mm baseline (mm)**				
Median (range)	0.8 (0.2–1.5)	0.5 (0.3–2)	0.6 (0.2–2)	0.744 (MW)
Mean ± SD	0.70 ± 0.41	0.77 ± 0.48	0.73 ± 0.44	
**Lingual STT 2 mm baseline (mm)**				
Median (range)	1.2 (0.2–2.5)	1 (0.3–2.1)	1.10 (0.2–2.50)	0.935 (MW)
Mean ± SD	1.33 ± 0.74	1.28 ± 0.55	1.31 ± 0.64	
**Clinical and radiographic outcomes**				
**Buccal KG at implant placement (mm)**				
Median (range)	5 (0–8)	5 (3–8)	5 (0–8)	0.806
Mean ± SD	4.90 ± 2.27	5.20 ± 1.47	5.05 ± 1.89	
**STT crestal at implant placement (mm)**				
Median (range)	3 (0.6–5)	2.5 (0.5–4.5)	3 (0.5–5)	0.325
Mean ± SD	3.04 ± 1.34	2.58 ± 1.09	2.81 ± 1.23	
**HRW at crest (mm)**				
**Median (range)**				
Before implant placement	7 (3–12)	6.5 (3–10)	6.75 (3–12)	1.000
After implant placement	8 (5–12)	7.5 (3–10)	8 (3–12)	0.983
Median of differences	0.5 (–0.5 to 2.5)	0 (–0.5 to 1.5)	0 (–0.5 to 2.5)	0.621
**Mean ± SD**				
Before implant placement	7.10 ± 2.44	6.93 ± 2.12	7.02 ± 2.25	
After implant placement	7.67 ± 2.03	7.39 ± 2.05	7.53 ± 2.01	
Mean differences	0.57 ± 0.88	0.46 ± 0.54	0.51 ± 0.74	
**HRW at 2 mm (mm)**				
**Median (range)**				
Before implant placement	8 (0–12)	8 (3–11)	8 (0–12)	0.935
After implant placement	8 (5–11.5)	8.1 (3–11)	8 (3–11.5)	0.813
Median of differences	0 (–1 to 7)	0 (–0.6 to 1)	0 (–1 to 7)	0.983
**Mean ± SD**				
Before implant placement SD	7.48 ± 3.1	7.84 ± 2.2	7.66 ± 2.65	
After implant placement	8.24 ± 2.07	8.28 ± 2.07	8.26 ± 2.04	
Mean differences	0.76 ± 1.91	0.44 ± 0.45	0.6 ± 1.41	
**Buccal bone thickness at 2 mm (in mm)**				
**Median (range)**				
Before implant placement	2 (0–5)	1.5 (0–3.5)	1.75 (0–5)	0.806
After implant placement	2 (0–5)	1.5 (0–3.5)	2 (0–5)	0.713
Median of differences	0 (−0.3 to 0.5)	0 (0–0.5)	0 (−0.3 to 0.5)	0.744
**Mean ± SD**				
Before implant placement	1.85 ± 1.24	1.70 ± 1.10	1.78 ± 1.16	
After implant placement	1.93 ± 1.29	1.73 ± 1.12	1.83 ± 1.19	
Mean differences	0.08 ± 0.23	0.03 ± 0.13	0.05 ± 0.19	
Palatal bone thickness at 2 mm (in mm)				
**Median (range)**				
Before implant placement	2 (1–3)	2 (1–2.5)	2 (1–3)	0.202
After implant placement	2 (0–3.5)	2 (1–3)	2 (0–3.5)	0.250
Median of differences	0 (−1 to 0.5)	0 (−0.5 to 1)	0 (−1 to 1)	0.683
**Mean** **±** S**D**				
Before implant placement	2.07 ± 0.78	1.73 ± 0.53	1.90 ± 0.67	
After implant placement	2.08 ± 0.98	1.77 ± 0.7	1.92 ± 0.85	
Mean differences	0.01 ± 0.33	0.04 ± 0.35	0.02 ± 0.34	
**CBCT measurements at 2 mm before ARP**				
**Median (range)**				
Before ARP at 2 mm	9.54 (7.5–12)	11.01 (7.65–13.55)	9.78 (7.5–13.55)	0.250
After ARP at 2 mm	8.35 (3.12–10.41)	8.60 (5.12–12.26)	8.56 (3.12–12.26)	0.744
Median of differences (range) SD	−0.99 (−6.54 to 0.27)	−1.51 (−5.74 to 0.06)	−1.12 (−6.54 to 0.27)	0.126
**Mean ± SD**				
Before ARP at 2 mm	9.48 ± 1.44	10.26 ± 1.76	9.87 ± 1.63	
After ARP at 2 mm	8.23 ± 1.9	8.52 ± 1.96	8.38 ± 1.9	
Mean differences	−1.25 ± 1.61	−1.74 ± 1.38	−1.49 ± 1.49	
**CBCT measurements at 4 mm before ARP**				
**Median (range)**				
Before ARP at 4 mm	10.11 (7.27–12.56)	10.54 (7.60–13.23)	10.18 (7.27–13.23)	0.285
After ARP at 4 mm	9.1 (6.63–12.07)	9.48 (6.79–12.33)	9.37 (6.63–12.33)	0.412
Median of differences (range)	−0.57 (−1.78 to 0.32)	−0.81 (−2.65 to 0)	−0.7 (−2.65 to 0.32)	0.250
**Mean ± SD**				
Before ARP at 4 mm	9.84 ± 1.65	10.52 ± 1.61	10.18 ± 1.64	
After ARP at 4 mm	9.26 ± 1.65	9.68 ± 1.49	9.47 ± 1.56	
Mean differences (range)	−0.58 ± 0.51	−0.84 ± 0.67	−0.71 ± 0.60	
**CBCT vertical changes after ARP**				
**Median (range)**				
Differences buccal vertical (range)	−0.87 (−1.94 to 3.06)	−1.07 (−4.31 to 5.23)	−0.93 (−4.31 to 5.23)	0.161
Differences palatal vertical (range)	−0.79 (−3.21 to 1.54)	−1.27 (−3.11 to 0.42)	−1.13 (−3.21 to 1.54)	0.250
**Mean ± SD**				
Differences buccal vertical	−0.48 ± 1.34	−1.07 ± 2.02	−0.77 ± 1.71	
Differences palatal vertical	−0.99 ± 1.08	−1.33 ± 0.73	−1.16 ± 0.92	

Abbreviations: ARP, alveolar ridge preservation, BT, bone thickness; CBCT, cone‐beam computed tomography; HRW, horizontal ridge width; KG, keratinized gingiva; MW, Mann–Whitney test; SD, standard deviation; STT, soft tissue thickness.

*Note*: Homogeneity of groups by demographic, clinical profile and linear measurements: Results of Chi‐2, Fisher's exact and Mann–Whitney's test. Homogeneity of groups by linear measurements and changes before/after IP: Results of Mann–Whitney's test. Homogeneity of groups by CBCT measurements and changes before/after ARP: Results of Mann–Whitney's test.

* *p* < 0.05.

#### 2.4.2 Wound healing and patient‐reported pain level after ARP and IP

After both ARP and IP surgical procedures, the wound healing index, adverse events, and pain questionnaire were evaluated. Healing progress was evaluated at 2 (± 3 days), 4 (± 3 days), and 12 (± 3 days) weeks (Figure 2L, M, N; Supplemental Figure S4); wound healing was assessed using Landry's healing index,^31^ which grades the wound on a scale of 1–5, where 1 indicates very poor healing, and 5 indicates excellent healing (Supplemental Table S2). Pain levels were assessed using a self‐report questionnaire both after extraction and after IP. Pain was evaluated over the first week using 3 different scales and the intake of painkillers (Supplemental Figures S10, Appendix).

#### 2.4.3 Impact of additional grafting on wound healing and PROs (please refer to the Appendix and supplemental Figure S9, supplemental Figure S10)

#### 2.4.4 Bone density (please refer to the Appendix and supplemental Table S10)

#### 2.4.5 Pink Esthetic Score (PES) (please refer to the Appendix and supplemental Figure S11)

#### 2.4.6 Overall patient satisfaction (please refer to the Appendix)

### 2.5 Sample Size

The sample size for this study was determined based on a power analysis conducted using data from Iasella et al. (2003),^10^ which measured vertical ridge height changes in the mid‐buccal location. The reported changes were 1.3 ± 2.0 mm for the RP group and −0.9 ± 1.6 mm for the extraction alone (EXT) group. From this, we calculated an effect size of d = 1.21. Based on this effect size, a sample size of at least 24 patients (12 per group) was required to detect the effect with 80% power and 95% confidence using a 2‐sample t‐test. To account for potential dropouts during the study, we added 3 additional patients to each group.

### 2.6 Statistical analysis

Statistical analysis involved descriptive statistics for categorical variables (frequencies) and continuous variables (mean, standard deviation [SD], range, median, percentiles) were computed using SPSS software. Nonparametric methods were used for non‐normally distributed data (Shapiro–Wilk test), with Mann–Whitney's test for group comparisons and Wilcoxon's test for intra‐group comparisons (Bonferroni correction). Ordinal pain questionnaire data were analyzed using the Brunner–Langer model for longitudinal data, assessing main effects and interactions with an analysis of variance (ANOVA) ‐type statistic. Chi‐squared and Fisher's exact tests evaluated categorical variable homogeneity, using a significance level of 5% (α = 0.05).

## 3 RESULTS

### 3.1 Participants

A total of 30 enrolled patients underwent ARP (One site per patient) before the IP. Three patients were excluded upon tooth extraction and before randomization due to bone dehiscence of more than 5 mm that was observed after extraction. When a patient was excluded after extraction, a new patient was recruited to maintain equal sample sizes in both groups. The study population comprised 18 males (60%) and 12 females (40%), with an average age of 58.2 ± 14.2 years (range: 24–77 years; Table 1). All 30 included participants completed the study at the point of IP. One patient in each group had 2 adjacent teeth extracted after inclusion, but only the first site was evaluated.

### 3.2 Baseline data

Eighty percent of extracted teeth were maxillary (60% premolars, 30% incisors, and 10% canines), with a consistent distribution across groups (Supplemental Table S3). There were no significant differences in baseline demographic data between groups regarding age or gender. At baseline, lingual bone thickness at 2 mm was significantly different between groups (*p* = 0.026), with medians of 0.80 mm in the control group and 1.50 mm in the test group.

### 3.3 Outcomes

#### 3.3.1 CBCT and clinical measurements of ridge width

##### 3.3.1.1 Clinical horizontal ridge width measurements at 2 mm

The median ridge width measured 2 mm below the crest before IP was similar in both groups, with the control group at 8.0 mm (mean 7.48 ± 3.10 mm) and the test group at 8.0 mm (mean 7.84 ± 2.20 mm).

##### 3.3.1.2 CBCT horizontal ridge width measurements at 2 mm

In the control group, ridge width significantly decreased from a median of 9.54 (mean 9.48 ± 1.44 mm) before ARP to 8.35 (mean 8.23 ± 1.88 mm) post‐ARP (*p* = 0.002). The test group also significantly reduced from median 11.01 (mean 10.26 ± 1.76 mm) to median 8.60 (mean 8.52 ± 1.96 mm, *p* = 0.002). The reduction was comparable between groups, with no significant difference (*p* = 0.126).

##### 3.3.1.3 CBCT horizontal ridge width measurements at 4 mm

In the control group, horizontal ridge width decreased from a median of 10.11 (mean 9.84 ± 1.65 mm) before ARP to median 9.10 (mean 9.26 ± 1.65 mm post‐ARP, *p* = 0.004). The test group showed a reduction from median 10.54 (mean 10.52 ± 1.61 mm) to 9.48 (mean 9.68 ± 1.49 mm, *p* = 0.002). Again, the reductions were comparable, with no significant difference between groups (*p* = 0.250).

##### 3.3.1.4 CBCT vertical bone changes

In the control group, the dimension decreased with a median change of −0.79 mm (mean −0.99 ± 1.08 mm), achieving statistical significance (*p* = 0.006). Similarly, the test group showed a significant reduction with a median change of −1.27 mm (mean −1.33 ± 0.73 mm, *p* < 0.001). The reductions were comparable between groups, with no significant difference observed (*p* = 0.250; Table 1).

The test and control groups showed similar mean values at both 2 mm and 4 mm levels, and the 95% confidence intervals for the between‐group differences included zero, indicating no statistically significant difference between them (Supplemental Table S4).

#### 3.3.2 CBCT and clinical measurements of soft tissue thickness (STT)

##### 3.3.2.1 Clinically measured vertical and facial STT

The clinical vertical STT measured at the time of IP was mean 3.04 ± 1.34 mm in the control group and 2.58 ± 1.09 mm in the test group. The soft tissue thickness measured 2 mm below the crest at the time of IP was mean 1.95 ± 1.61 mm in the control group and 1.77 ± 1.00 mm in the test group.

##### 3.3.2.2 Digitally measured vertical STT

STT was quantified at the midpoint of the alveolar crest using superimposed CBCT and intraoral scans (IOS), revealing no significant differences between the control (mean STT: 2.67 ± 0.69 mm) and test groups (mean STT: 2.13 ± 0.80 mm, *P* = 0.325). Additionally, no significant differences were noted between clinical and CBCT measurements (Supplemental Table S5 and supplemental Figure S3).

#### 3.3.3 Comparison between maxillary and mandibular outcomes

Adding an ACM over the collagen plug during ARP did not show statistically significant differences compared with the control group across clinical, radiographic, or pain‐related outcomes. Both at ARP and at IP group analyses revealed similar changes in ridge width, buccal and palatal bone thickness, soft tissue dimensions, and wound healing index. CBCT measurements confirmed comparable dimensional changes between groups, except for a slightly greater buccal bone loss in the maxillary test group (−1.38 mm vs. −0.55 mm, *p* = 0.002, Supplemental Table S6), however no differences were observed in the mandible. Pain levels and analgesic consumption decreased over time in both groups, with only minor arch‐dependent variations and no statistically significant differences. Implant characteristics, surgical parameters, and postoperative healing were consistent across groups and arches.

STT at the crestal and 2 mm levels was evaluated for both the maxilla and mandible in control and test groups. At the crestal level, STT was slightly higher in the maxilla than in the mandible for both groups, with minimal differences between control and test (maxilla: 3.09 mm vs. 2.60 mm; mandible: 2.83 mm vs. 2.50 mm). At 2 mm below the crest, the maxilla exhibited thinner soft tissue in the test group compared to control (1.73 mm vs. 2.12 mm), whereas in the mandible, the test group showed slightly thicker tissue than control (1.90 mm vs. 1.27 mm Supplemental Table S7). Overall, the control group revealed greater STT in the maxilla than the mandible at both levels. This indicates that dimensional changes after ARP were more pronounced in the maxilla, while the mandible maintained more stable ridge dimensions.

#### 3.3.4 Wound healing index after ARP and IP

Landry's healing index improved significantly over time (2‐, 4‐, and 12‐weeks post‐surgery) (*p* < 0.001) but showed no significant differences between groups (*p* = 0.260). At 4 weeks after extractions, the BACM test group showed slightly better results, though differences remained nonsignificant (Supplemental Figure S5).

Two weeks after IP, 66.7% of the test group achieved a wound healing index of 5 (excellent), compared to 46.7% in the control group, which had 13% with poor healing (Grade 2). Despite the test group's descriptively better outcomes, the difference was not statistically significant (*p* = 0.305, Mann–Whitney U test; Supplemental Figure S5).

## 4 DISCUSSION

Following tooth extraction, the alveolar bone undergoes a sequence of biological events involving both bone formation and resorption, resulting in dimensional and morphological alterations of the alveolar ridge (Sculean, Stavropoulos, & Bosshardt, 2019).^32^

To reduce these dimensional changes, ARP techniques using different grafting materials have been developed, although their results vary across studies. According to a systematic review and meta‐analysis conducted by Avila‐Ortiz et al., the process of ARP through socket grafting has been shown to potentially reduce horizontal bone loss by 1.5–2.4 mm, vertical mid‐buccal bone loss by 1–2.5 mm, and mid‐lingual vertical bone loss by 0.8–1.5 mm when compared to simple tooth extraction without preservation techniques (Avila‐Ortiz et al. 2020).^33^ Couso‐Queiruga et al. (2023)^2^ reported progressive increases in mineralized tissue with DBBM with collagen (DBBM‐C) and collagen membranes, reaching 37% after 9 months. Without ARP, ridge width and height commonly decrease by 3.9 mm and 1.7 mm, respectively (van der Weijden et al., 2009),^34^ whereas ARP can reduce these losses by 1.5–2.5 mm (Avila‐Ortiz et al., 2020).^33^ Jambhekar et al. (2015)^35^ further demonstrated 35.7% vital bone and minimal ridge width reduction (1.3 mm) following flapless socket grafting, underscoring the benefit of grafting procedures in preserving ridge dimensions.

Araujo and colleagues documented DBBM‐C dynamics in an experimental dog study.^5^ The authors reported that bone formation was faster in apical and lateral portions of the socket compared to central and marginal areas. Due to prolonged resorption rate of xenograft, the study revealed that DBBM‐C for ARP may be associated with delayed post‐extraction sites healing, particularly in the central and coronal parts of the extraction site. Lindhe and colleagues did another study utilizing a xenograft in extraction sockets.^36^ Patients were allocated into 2 groups: a test group with DBBM‐C and a control group without grafting. The results revealed that the amount of xenograft surrounded by new bone formation increases with longer healing time.

In alignment with these findings, our study showed no statistically significant differences between the DBBM‐C and control groups in clinical outcomes at 4 months. Both groups demonstrated comparable levels of new bone formation, residual graft particles, and connective tissue (Supplemental Table S8). These results parallel those of Couso‐Queiruga et al. (2023),^2^ whose core biopsies were analyzed within a similar 3‐ to 9‐month healing period.

Although no statistically significant differences were observed between groups, the BACM group exhibited a greater horizontal ridge reduction by more than 1 mm compared to the control. Clinically, this may be relevant, as previous studies indicate that a buccal bone thickness (BBT) of ≥2 mm is critical to prevent additional grafting. The potential explanation is that the test group required additional soft tissue elevation/ tunneling for membrane placement, which may have caused minor surgical trauma and contributed to slightly greater resorption. Therefore, even in the absence of statistical significance, this finding may have practical implications for clinicians considering the use of BACM in ARP.

Wound healing progression and patient‐reported pain levels were slightly more favorable in the BACM group, without significant intergroup differences (Supplemental Figure S6 and S7).

This study had several limitations. To minimize anatomical variability, the study focused on Type 1 and Type 2A extraction sockets with intact bony walls, excluding those with more than 5 mm of buccal bone dehiscence.^27^ This may have hindered the detection of statistically significant differences in ridge width between groups.^27^ However, the extraction was done in a flapless fashion in both groups. Although the tunneling method for placing the membrane on the facial aspect of the socket involves minimal tissue elevation similar to flap reflection, its influence on facial bone remodeling still requires further study (Saleh et al., 2022).^37^

The exclusion of molars from the evaluation could be viewed as a limitation of the study. However, the focus was on the aesthetic zone and how the addition of a membrane may influence the outcomes. A separate study could be conducted in the future to specifically evaluate molars.

Future research should include long‐term clinical trials to evaluate the sustainability of bone and tissue preservation with BACM.

## 5 CONCLUSION

The addition of a BACM did not lead to significant improvements in mean horizontal or vertical bone gain, nor did it have a measurable effect on soft tissue outcomes during ARP. Although no statistically significant differences were observed, a clinically relevant effect remains possible. PROs were also not statistically significant between the groups. Therefore, no superiority of the BACM treatment over the control group was demonstrated. From a health economics standpoint, rejecting the more costly option when no significant differences exist is a reasonable approach.

## AUTHOR CONTRIBUTIONS

**Magdalena Orłowska**: Conception; supervision, and manuscript draft. **Muhammad Saleh**: Surgical procedures; project supervision, and manuscript review. **Ann Decker**: Histological and immunohistological analysis and interpretation. **Junying Li**: Prosthetic rehabilitation and supervision. **Alice Ou**: Project administration. **Nataly Zambrana**: Radiographic analysis and data curation. **Paola Nava**: Radiographic analysis; visualization; data curation, and writing‐revision. **Fernanda Deller**: Statistical analysis. **Hom‐Lay Wang**: Surgical procedures; supervision, and administration.

## CONFLICT OF INTEREST STATEMENT

The authors declare no conflicts of interest.

## Supporting information



Supporting Information

**Supplemental Figure 1**: Flow chart of time points for data collection. Abbreviations: FU, follow‐up; ARP, alveolar ridge preservation.
**Supplemental Figure 2**: Clinical measurements after extraction. (A–C) Buccal and lingual bone thickness and ridge width were measured using a caliper 2 and 4 mm below the crest after subtracting the previously measured tissue thickness; (D–F) Keratinized tissue width and papilla height was measured on the buccal site using a periodontal probe (UNC Probe).
**Supplemental Figure 3**: Soft tissue measurements. Soft tissue thickness was assessed through linear measurements of the second CBCT superimposed with intraoral scans, and all were analyzed using the BlueSky Plan.
**Supplemental Figure 4**: Clinical photos. 2, 4 and 12 weeks follow up; (A–F) control group; (G–L) test group; (A) 2 weeks FU CG occlusal view, (B) 2 weeks FU CG lateral view, (C) 4 weeks FU CG occlusal view, (D) 4 weeks FU CG lateral view, (E) 12 weeks FU CG occlusal view, (F) 12 weeks FU CG lateral view, (G) 2 weeks FU TG occlusal view, (H) 2 weeks FU TG lateral view, (I) 4 weeks FU TG occlusal view, (J) 4 weeks FU TG lateral view, (K) 12 weeks FU TG occlusal view, (L) 12 weeks FU TG lateral view. Abbreviations: FU, follow‐up; CG, control group; TG, test group.
**Supplemental Figure 5: A)** Changes in wound healing index by group 2,4 and 12 weeks after extraction. Wound healing was assessed using Landry's healing index at 2‐, 4‐, and 12‐weeks post‐surgery. The index improved significantly over time (*p* < 0.001) but showed no significant differences between groups (*p* = 0.260). At 4 weeks after extractions, the Amnion chorion membrane (ACM) test group showed slightly better results, though differences remained nonsignificant. **B)** Changes in wound healing index by group 2 weeks after implant placement. Wound healing index measured by Landry wound healing index. Two weeks post‐implant placement, in the test group, 66.7% achieved a wound healing index of 5 (excellent), compared to 46.7% in the control group, which had 13% with poor healing (Grade 2). Despite descriptively better outcomes in the test group, the difference was not statistically significant (*p* = 0.305, Mann–Whitney U test).
**Supplemental Figure 6A**. Pain: Verbal scale Day 1 to 7 after ARP by group.
**Supplemental Figure 6 B**. Number of painkillers per group after ARP.
**Supplemental Figure 7A**. Pain assessment/ line scale.
**Supplemental Figure 7 B**. Relative effect plot of the numbers of painkillers after IP per group.
**Supplemental figure 8**: (A) **Alveolar ridge before implant placement, reentry procedure**. (B) Full‐thickness mucoperiosteal flaps. (C) An analog modular surgical guide was created to guide a 2.75 mm trephine, which was used to obtain a bone core biopsy at the planned implant site. (D) Alveolar ridge after core biopsy was completed.
**Supplemental Figure 9**. Wound healing index at 2 weeks after implant placement by additional grafting (Mann–Whitney's test).
**Supplemental Figure 10**. Painkillers were taken after additional grafting, and the patient reported verbal pain. (A) Painkillers taken. Comparison between additional grafting vs. no additional grafting patients, (B) Patient‐reported verbal pain scale. Comparison between additional grafting vs. no additional grafting patients, (C) Patient‐reported verbal pain scale. Comparison between additional grafting vs. no additional grafting in control and test groups.
**Supplemental Figure 11**. Pink Esthetic Score by group.

Supporting Information
