# Supplementary material for: The effect of bioactive membrane in alveolar ridge preservation: A double‐blind randomized clinical trial
Source: J Periodontol. 2026 Feb 26;97(9):1871–84. doi: 10.1002/jper.70094 (PMC13568902; doi:10.1002/jper.70094)
Supplement: Supplementary file 1 [file JPER-97-1871-s001.docx]

**Supplemental table 1.** Chronological outcome assessment

| **Timeline** | **Outcome** | **Means of measurement** | | **Measurement unit** | **Assessor** | **Rank of outcome** | **Prerequisite by BA-COSM: Rank∘** |
| --- | --- | --- | --- | --- | --- | --- | --- |
| **Before extraction** | Horizontal ridge dimensions at 2 & 4 mm | CBCT | | mm | Blinded assessor A | Primary | YES: Mandatory |
|  | Vertical ridge dimensions at 2 & 4 mm |  |  |  |  | Secondary |  |
| **At time of extraction & ARP** | KM Width | Periodontal probe | | mm | Surgeon | Secondary | NO |
|  | Buccal tissue thickness at 2 & 4 mm | Endodontic file | | mm | Surgeon | Secondary | NO |
|  | Horizontal ridge dimensions at 2 & 4 mm | Caliper | | mm | Surgeon | Primary | NO |
| **After ARP** | Wound Healing | Landry's healing index | Objective score (1-4) by assessor | Blinded assessor B | Secondary | | YES: Mandatory |
|  | Post-Operative Pain | Line scale | Subjective score | Patient | Secondary | | YES: Mandatory |
|  | Post-Operative Pain | Box scale | Subjective score (0-10) | Patient | Secondary | | YES: Mandatory |
|  | Post-Operative Pain | Verbal rating scales | Subjective score (no pain-extremely painful) | Patient |  | | YES: Mandatory |
| **Before implant surgery** | Horizontal and vertical ridge dimensions at 2 & 4 mm | CBCT | mm | Blinded assessor A | Primary | | YES: Mandatory |
| **Implant placement** | KM Width | Periodontal probe | mm | Surgeon | Secondary | | NO |
|  | Buccal tissue thickness at 2 & 4 mm | file | Endodontic mm | Surgeon | Secondary | | NO |
|  | Crestal tissue thickness | Endodontic file | mm | Surgeon | Secondary | | NO |
|  | Horizontal ridge dimensions at 2 & 4 mm | Caliper | mm | Surgeon | Primary | | YES: Mandatory |
|  | Buccal bone thickness before and after implant placement | Caliper | mm | Surgeon | Secondary | | YES: Mandatory |
|  | Palatal bone thickness immediately before and after implant placement | Caliper | mm | Surgeon | Secondary | | NO |
|  | Bone density | Subjective according to Misch et al. bone density classification | D1-D4 | Surgeon | Secondary | | NO |
|  | Ability to place the implant in a prosthetically guided implant position | Objective according to the previously reported prosthetic and biologic guidelines ^33^ | Binary (Yes/No) | Blinded assessor A | Secondary | | YES: Mandatory |
|  | Need for additional grafting | If buccal bone thickness is < 2mm after implant placement in the ideal prosthetic position | Binary (Yes/No) | Surgeon | Secondary | | NO* |

Abbreviations: CBCT, Cone beam computed tomography: ARP, alveolar ridge preservation: KM, keratinized mucosa; H&M, hematoxylin, and eosin.

∘ All mandatory and optional domains, as per BA-COSM, were reported. Those marked No were additional outcomes considered valuable for assessing the efficacy of adding a BACM.
* The need for additional grafting was neither considered a mandatory nor optional domain. However, the consensus report mentioned that “in staged BA procedures (like ARP), the ability or not to place the implant in a prosthetically guided implant position with the endosteal portion of the implant completely in bone with more than 1–1.5 mm thickness on the buccal and oral aspect. Investigators should also report the need for additional BA based on the previous objective”. The authors used a different threshold for the buccal/oral bone thickness (≥2 mm).

**Supplemental table 2.** Wound Healing Index by Landry et al. 1988

| 1 Very Poor | Tissue color: ≥50% of gingiva red  Response to palpation: Bleeding  Granulation tissue: Present  Incision margin: Not epithelialized, with loss of  epithelium beyond incision margin  Suppuration: Present |
| --- | --- |
| 2 Poor | Tissue color: ≥50% of gingiva red  Response to palpation: Bleeding  Granulation tissue: Present  Incision margin: Not epithelialized, with  connective tissue exposed |
| 3 Good | Tissue color: ≥25% and<50% of gingiva red  Response to palpation: No bleeding  Granulation tissue: None  Incision margin: No connective tissue exposed |
| 4 Very Good | Tissue color: <25% of gingiva red  Response to palpation: No bleeding  Granulation tissue: None  Incision margin: No connective tissue exposed |
| 5 Excellent | Tissue color: All tissues pink  Response to palpation: No bleeding  Granulation tissue: None  Incision margin: No connective tissue exposed |

**Supplemental Table 3.** Extracted tooth number and arch position by group

|  | **Group** | | | | | |
| --- | --- | --- | --- | --- | --- | --- |
|  | **Total** | | **Control** | | **Test** | |
|  | **N** | **%** | **N** | **%** | **N** | **%** |
| **Total** | 30 | 100.0 | 15 | 100.0 | 15 | 100.0 |
| **I** | 9 | 30.0 | 4 | 26.7 | 5 | 33.3 |
| **C** | 3 | 10.0 | 2 | 13.3 | 1 | 6.7 |
| **PM** | 18 | 60.0 | 9 | 60.0 | 9 | 60.0 |
| **Maxilla** | 24 | 80 | 12 | 80 | 12 | 80 |
| **Mandible** | 6 | 20 | 3 | 20 | 3 | 20 |

Abbreviations: I, incisor; C, canine: PM, premolar, N, numbers

**Supplemental table 4.**

|  | | | | | | | | | | | | | | | | | **Shapiro-Wilk** | | | |
| --- | --- | --- | --- | --- | --- | --- | --- | --- | --- | --- | --- | --- | --- | --- | --- | --- | --- | --- | --- | --- |
|  | | **N** | |  | | **Mean** | | **Median** | | | **SD** | | **Minimum** | | **Maximum** | | **W** | | **p** | |
| Test before ARP at 2 mm |  | 15 |  |  |  | | 10.259 |  | 11.010 |  | 1.762 |  | 7.6500 |  | 13.55 |  | 0.927 |  | 0.250 |  |
| Test after ARP at 2 mm |  | 15 |  |  |  | | 8.521 |  | 8.600 |  | 1.963 |  | 5.1200 |  | 12.26 |  | 0.950 |  | 0.517 |  |
| Differences at 2 mm |  | 15 |  |  |  | | 1.739 |  | 1.510 |  | 1.380 |  | -0.0600 |  | 5.74 |  | 0.858 |  | 0.023 |  |
| Test before ARP at 4 mm |  | 15 |  |  |  | | 10.524 |  | 10.540 |  | 1.605 |  | 7.6000 |  | 13.23 |  | 0.964 |  | 0.764 |  |
| Test after ARP at 4 mm |  | 15 |  |  |  | | 9.680 |  | 9.480 |  | 1.490 |  | 6.7900 |  | 12.33 |  | 0.986 |  | 0.995 |  |
| Differences at 4 mm |  | 15 |  |  |  | | 0.844 |  | 0.810 |  | 0.666 |  | 0.0000 |  | 2.65 |  | 0.897 |  | 0.084 |  |

|  | | | | | | | | | | | | | | | | | |
| --- | --- | --- | --- | --- | --- | --- | --- | --- | --- | --- | --- | --- | --- | --- | --- | --- | --- |
|  | | | | | | | | | | | | | | **Shapiro-Wilk** | | | |
|  | | **N** | | **Mean** | | **Median** | | **SD** | | **Minimum** | | **Maximum** | | **W** | | **p** | |
| Control before ARP at 2 mm |  | 15 |  | 9.478 |  | 9.540 |  | 1.438 |  | 7.500 |  | 12.00 |  | 0.941 |  | 0.393 |  |
| Control after ARP at 2 mm |  | 15 |  | 8.232 |  | 8.350 |  | 1.883 |  | 3.120 |  | 10.41 |  | 0.886 |  | 0.059 |  |
| Differences at 2 mm |  | 15 |  | 1.246 |  | 0.990 |  | 1.606 |  | -0.270 |  | 6.54 |  | 0.684 |  | < .001 |  |
| Control before ARP at 4 mm |  | 15 |  | 9.841 |  | 10.110 |  | 1.649 |  | 7.270 |  | 12.56 |  | 0.964 |  | 0.758 |  |
| Control after ARP at 4 mm |  | 15 |  | 9.262 |  | 9.100 |  | 1.651 |  | 6.630 |  | 12.07 |  | 0.948 |  | 0.501 |  |
| Differences at 4 mm |  | 15 |  | 0.579 |  | 0.570 |  | 0.506 |  | -0.320 |  | 1.78 |  | 0.973 |  | 0.905 |  |

**Supplemental table 5.** Soft tissue thickness comparison using clinical measurements and CBCT with intraoral scan superimposition technique using Shapiro-Wilk.

| Soft tissue thickness at crest 4 months after extraction (mm) | Groups N Median Mean SD Minimum Maximum |
| --- | --- |
| CBCT/Intraoral scan | Control 15 2.56 2.67 0.69 1.7 3.89  Test 15 1.99 2.13 0.80 0.9 3.87 |
| Clinical measurement | Control 15 3.0 3.04 1.34 0.6 5.0  Test 15 2.5 2.58 1.09 0.5 4.5 |

The Shapiro-Wilk test was performed to verify the normality of the data. Both groups had a normal distribution (control group p=0.37; test group p=0.84). Therefore, the Student’s test was performed to compare the test and control group.

**Supplemental Table 6**. CBCT: Changes in buccal and palatal vertical cortical plate before/after ARP by group and arch

|  | | **Arch** | | | | | | | | |
| --- | --- | --- | --- | --- | --- | --- | --- | --- | --- | --- |
|  |  | **Total** | | | **maxilla** | | | **mandible** | | |
|  |  | **Group** | | | **Group** | | | **Group** | | |
|  |  | **Total** | **control** | **test** | **Total** | **control** | **test** | **Total** | **control** | **test** |
| **DIFF Buccal vertical** | **N** | 30 | 15 | 15 | 24 | 12 | 12 | 6 | 3 | 3 |
|  | **Mean** | -.77 | -.48 | -1.07 | -.96 | -.55 | -1.38 | -.01 | -.20 | .18 |
|  | **Standard Deviation** | 1.71 | 1.34 | 2.02 | 1.22 | 1.26 | 1.08 | 3.04 | 1.93 | 4.39 |
|  | **Minimum** | -4.31 | -1.94 | -4.31 | -4.31 | -1.94 | -4.31 | -2.63 | -1.88 | -2.63 |
|  | **Maximum** | 5.23 | 3.06 | 5.23 | 3.06 | 3.06 | -.46 | 5.23 | 1.91 | 5.23 |
|  | **Percentile 25** | -1.67 | -1.29 | -2.07 | -1.31 | -1.20 | -1.68 | -2.07 | -1.88 | -2.63 |
|  | **Median** | -.93 | -.87 | -1.07 | -.93 | -.90 | -1.01 | -1.26 | -.63 | -2.07 |
|  | **Percentile 75** | -.50 | -.23 | -.62 | -.51 | -.32 | -.70 | 1.91 | 1.91 | 5.23 |
| **DIFF Palatal vertical** | **N** | 30 | 15 | 15 | 24 | 12 | 12 | 6 | 3 | 3 |
|  | **Mean** | -1.16 | -.99 | -1.33 | -1.21 | -1.01 | -1.40 | -.96 | -.90 | -1.03 |
|  | **Standard Deviation** | .92 | 1.08 | .73 | 1.01 | 1.20 | .77 | .44 | .38 | .58 |
|  | **Minimum** | -3.21 | -3.21 | -3.11 | -3.21 | -3.21 | -3.11 | -1.57 | -1.26 | -1.57 |
|  | **Maximum** | 1.54 | 1.54 | -.42 | 1.54 | 1.54 | -.49 | -.42 | -.51 | -.42 |
|  | **Percentile 25** | -1.57 | -1.56 | -1.66 | -1.76 | -1.75 | -1.76 | -1.26 | -1.26 | -1.57 |
|  | **Median** | -1.13 | -.79 | -1.27 | -1.20 | -.79 | -1.30 | -1.01 | -.92 | -1.10 |
|  | **Percentile 75** | -.53 | -.50 | -.54 | -.54 | -.46 | -.85 | -.51 | -.51 | -.42 |

**Supplemental Table** 7. Buccal side KG and STT at implant placement (IP) by group and arch

|  | | **Arch** | | | | | | | | |
| --- | --- | --- | --- | --- | --- | --- | --- | --- | --- | --- |
|  | | **Total** | | | **maxilla** | | | **mandible** | | |
|  | | **Group** | | | **Group** | | | **Group** | | |
|  | | **Total** | **control** | **test** | **Total** | **control** | **test** | **Total** | **control** | **test** |
| **Buccal side KG IP** | **N** | 30 | 15 | 15 | 24 | 12 | 12 | 6 | 3 | 3 |
|  | **Mean** | 5.05 | 4.90 | 5.20 | 5.48 | 5.46 | 5.50 | 3.33 | 2.67 | 4.00 |
|  | **Standard Deviation** | 1.89 | 2.27 | 1.47 | 1.66 | 1.92 | 1.45 | 1.86 | 2.52 | 1.00 |
|  | **Minimum** | .00 | .00 | 3.00 | 3.00 | 3.00 | 3.00 | .00 | .00 | 3.00 |
|  | **Maximum** | 8.00 | 8.00 | 8.00 | 8.00 | 8.00 | 8.00 | 5.00 | 5.00 | 5.00 |
|  | **Percentile 25** | 3.50 | 3.00 | 4.00 | 4.00 | 3.25 | 4.50 | 3.00 | .00 | 3.00 |
|  | **Median** | 5.00 | 5.00 | 5.00 | 6.00 | 6.00 | 5.50 | 3.50 | 3.00 | 4.00 |
|  | **Percentile 75** | 6.00 | 7.00 | 6.00 | 7.00 | 7.00 | 6.50 | 5.00 | 5.00 | 5.00 |
| **STT crestal IP** | **N** | 30 | 15 | 15 | 24 | 12 | 12 | 6 | 3 | 3 |
|  | **Mean** | 2.81 | 3.04 | 2.58 | 2.85 | 3.09 | 2.60 | 2.67 | 2.83 | 2.50 |
|  | **Standard Deviation** | 1.23 | 1.34 | 1.09 | 1.34 | 1.47 | 1.21 | .61 | .76 | .50 |
|  | **Minimum** | .50 | .60 | .50 | .50 | .60 | .50 | 2.00 | 2.00 | 2.00 |
|  | **Maximum** | 5.00 | 5.00 | 4.50 | 5.00 | 5.00 | 4.50 | 3.50 | 3.50 | 3.00 |
|  | **Percentile 25** | 2.00 | 2.00 | 2.00 | 2.00 | 2.00 | 1.75 | 2.00 | 2.00 | 2.00 |
|  | **Median** | 3.00 | 3.00 | 2.50 | 3.00 | 3.50 | 2.75 | 2.75 | 3.00 | 2.50 |
|  | **Percentile 75** | 4.00 | 4.00 | 3.50 | 4.00 | 4.00 | 3.50 | 3.00 | 3.50 | 3.00 |
| **STT 2 mm IP** | **N** | 30 | 15 | 15 | 24 | 12 | 12 | 6 | 3 | 3 |
|  | **Mean** | 1.86 | 1.95 | 1.77 | 1.93 | 2.12 | 1.73 | 1.58 | 1.27 | 1.90 |
|  | **Standard Deviation** | 1.32 | 1.61 | 1.00 | 1.40 | 1.76 | .96 | 1.03 | .64 | 1.39 |
|  | **Minimum** | .60 | .60 | .80 | .60 | .60 | .80 | .80 | .80 | 1.00 |
|  | **Maximum** | 6.50 | 6.50 | 4.00 | 6.50 | 6.50 | 4.00 | 3.50 | 2.00 | 3.50 |
|  | **Percentile 25** | 1.00 | 1.00 | 1.00 | 1.00 | 1.00 | 1.00 | 1.00 | .80 | 1.00 |
|  | **Median** | 1.35 | 1.50 | 1.20 | 1.50 | 1.50 | 1.55 | 1.10 | 1.00 | 1.20 |
|  | **Percentile 75** | 2.00 | 2.00 | 2.50 | 2.25 | 2.50 | 2.25 | 2.00 | 2.00 | 3.50 |

**Supplemental table 8**. Diameter by group

|  | **Group** | | | | | |
| --- | --- | --- | --- | --- | --- | --- |
|  | **Total** | | **control** | | **test** | |
|  | **N** | **%** | **N** | **%** | **N** | **%** |
| **Total** | 30 | 100.0 | 15 | 100.0 | 15 | 100.0 |
| **3,30** | 5 | 16.7 | 3 | 20.0 | 2 | 13.3 |
| **3,80** | 16 | 53.3 | 8 | 53.3 | 8 | 53.3 |
| **4,20** | 9 | 30.0 | 4 | 26.7 | 5 | 33.3 |

**Supplemental table 9.** Length by group

|  | **Group** | | | | | |
| --- | --- | --- | --- | --- | --- | --- |
|  | **Total** | | **control** | | **test** | |
|  | **N** | **%** | **N** | **%** | **N** | **%** |
| **Total** | 30 | 100.0 | 15 | 100.0 | 15 | 100.0 |
| **9,0** | 5 | 16.7 | 3 | 20.0 | 2 | 13.3 |
| **10,0** | 1 | 3.3 | 0 | .0 | 1 | 6.7 |
| **10,5** | 9 | 30.0 | 5 | 33.3 | 4 | 26.7 |
| **12,0** | 15 | 50.0 | 7 | 46.7 | 8 | 53.3 |

**Supplemental table 10.** Bone density by group

|  | **Group** | | | | | |
| --- | --- | --- | --- | --- | --- | --- |
|  | **Total** | | **control** | | **test** | |
|  | **N** | **%** | **N** | **%** | **N** | **%** |
| **Total** | 30 | 100.0 | 15 | 100.0 | 15 | 100.0 |
| **2** | 5 | 16.7 | 2 | 13.3 | 3 | 20.0 |
| **3** | 15 | 50.0 | 8 | 53.3 | 7 | 46.7 |
| **4** | 9 | 30.0 | 4 | 26.7 | 5 | 33.3 |
| **5** | 1 | 3.3 | 1 | 6.7 | 0 | .0 |

**APPENDIX**

## Appendix A: Schedule of Events Table

| VISIT | Screening (V1) | Baseline  Socket Surgery (V2) | Post-op (V3, V4, V5) | Implant Surgery & Biopsy (V6) | Post-op (V7) | Stage II surgery  (V8) | Post – op  Crown impression  (V9) | Crown delivery  (V10) | Follow-up  (V11) |
| --- | --- | --- | --- | --- | --- | --- | --- | --- | --- |
| Timeline |  | Day 0 | 2 (±3 days), 4 (±3 days), & 12 (±3 days) weeks | 4 months  (±2 weeks) | 2 weeks (±3 days) after V6 | 4 months  (±2 weeks)  after V6 | 2 weeks  (±2 weeks)  after V8 | 2-3 weeks (±3 days)  after V9 | 6 months (±2 weeks)  after V10 |
| Informed consent and confirm eligibility | X |  |  |  |  |  |  |  |  |
| Radiograph(s) | X |  |  | X |  | X |  | X | X |
| Impressions | X |  | X* | X |  | X | X |  |  |
| Cone-beam CT | X |  |  | X |  |  |  |  |  |
| Socket augmentation |  | X |  |  |  |  |  |  |  |
| Pain survey |  | X |  | X |  |  |  |  |  |
| Wound healing index (WHI) |  |  | X |  | X |  |  |  |  |
| Dental Implant placement |  |  |  | X |  |  |  |  |  |
| Implant crown placement |  |  |  |  |  |  | X |  |  |
| Clinical measures |  | X |  | X |  | X |  | X | X |
| Oral exam and Photographic records | X | X | X | X | X | X | X | X | X |

* V- visits; At 12 weeks (V5) visit only

## Appendix B: Rating Scales for Pain Measurement

Subject: ________________________	Date: __________________

**Rating Scales for Pain Measurement**

**Line Scale:**

Please rate the level of your pain by placing an “X” on the following line.

**No Pain	Worst pain imaginable**

**Box Scale:**

Rate the level of your pain by circling one number on the scale, where 0 means “no pain” and 10 means “worst pain imaginable.”

| 0 | 1 | 2 | 3 | 4 | 5 | 6 | 7 | 8 | 9 | 10 |
| --- | --- | --- | --- | --- | --- | --- | --- | --- | --- | --- |

**Verbal Rating Scale:**

Circle the phrase that best describes your pain

| No Pain | Slightly Painful | Moderately Painful | Very Painful | Extremely Painful |
| --- | --- | --- | --- | --- |

**How much ibuprofen (or other painkiller) did you take today? ______**
