# Supplementary material for: The effect of bioactive membrane in alveolar ridge preservation: A double‐blind randomized clinical trial
Source: J Periodontol. 2026 Feb 26;97(9):1871–84. doi: 10.1002/jper.70094 (PMC13568902; doi:10.1002/jper.70094)
Supplement: Supplementary file 2 [file JPER-97-1871-s002.docx]

# 2 **| MATERIAL AND METHODS**

### **2.3 Interventions**

### **2.3.2. Implant placement (IP) procedures**.

Reentry procedures were performed 4 months (±2 weeks) after ARP to place a dental implant. Prior to the surgical intervention, a new standardized radiograph, digital impression, CBCT scan, and surgical guides were prepared (Figure 2 o, Supplemental figure 8). All implant surgical procedures were performed under local anesthesia by two calibrated surgeons (HLW and MS). Full-thickness mucoperiosteal flaps were elevated on the buccal and palatal sides to access the residual ridge, do clinical measurements. Soft tissue thickness and ridge width were measured at 2 mm and 4 mm below the crest using a caliper (Iwanson; Hu-Friedy, USA) (Supplemental figure 10). Soft tissue thickness at the crest was measured after flap elevation using a perpendicular incision to standardize the process and minimize measurement variability.

Based on the digital scan performed before the re-entry surgery, an analog modular surgical guide was created to guide a 2.75 mm trephine, which was used to obtain a bone core biopsy at the planned implant site (Figure 2 o, p; Supplemental figure 8). The same modular guide was used later for implant placement according to the manufacturer’s recommendations (Fusion Implants, Biohorizons, Birmingham, Alabama, USA; Figure 2q).

Standardized PA radiographs confirmed pin positioning during surgery. Regular platform implants were placed, all using a two-stage approach, ensuring a minimum insertion torque of >30 N/cm² for primary stability. Implants were placed 4 mm from the future gingival margin of the digitally planned crown, with diameters of 3.3 mm, 3.8 mm, or 4.2 mm, and lengths of 9, 10.5, or 12 mm, depending on the tooth site being replaced and the space available. An additional bone graft was added if the buccal bone thickness was found to be <2 mm (Monje et al., 2019; Spray, Black, Morris, & Ochi, 2000). All implants achieved primary stability, evaluated by torque testing at 35 N/cm². Tactile assessment of bone density during surgery provided a clinical evaluation of bone quality. The surgical site was sutured for complete closure using 3/0 PTFE sutures. Depending on the buccal bone thickness at the platform level, some patients required additional bone grafting at the time of implant placement if the buccal bone thickness was less than 2 mm. Scientific evidence supports the importance of buccal bone thickness (BBT) for implant stability.

### **2.3.3. Stage-II surgery, follow-up evaluation**

Approximately four months after implant placement, a healing abutment was placed (Figure 2 r). No buccal bone dehiscence was observed during stage-II surgery in any of the cases. Two to three weeks later, an impression was taken to create a custom-made crown. The stability of dental implants under function was evaluated over a period of approximately 6 months following placement. A standardized periapical radiograph was taken before the restorative phase to assess implant integration. Implants showing mobility, pain, suppuration, significant bleeding, or radiographic signs of failure were classified as failures. Implants were restored within 4 months of placement, and both implants and restorations were monitored for an additional 6 months.

#### **2.4 Outcomes**

Although our study began in late 2022, before the publication of the ID-COSM and the BA-COSM guidelines, we included all the mandatory and optional aspects required and recommended later by these guidelines in March 2023. Due to the timing discrepancy between the release of the guidelines and the initiation of our study, we could not strictly adhere to all aspects at the time of registration on ClinicalTrials.gov.

2.4.1. **Horizontal and vertical hard and soft tissue outcomes after ARP**

A) **CBCT measurements**

All patients underwent two CBCT scans at two time points: before extraction and four months post-extraction, using a 3D Accuitomo machine (J. Morita Corp, Japan). The baseline CBCT assessed the tooth position, horizontal bone thickness, dehiscence, and surrounding anatomical structures. The post-extraction CBCT evaluated the site for implant placement. To assess horizontal and vertical bone changes, automatic segmentation of the full arch and point-based alignment using the implant planning software Blueskyplan v.4.12.13 (Blueskybio, USA) was performed and blindly assessed by the same investigator (NZ). Baseline and four months after extraction CBCT were automatically segmented as bone surfaces and exported as STL (standard triangulation language) files. A mid-sagittal slice representative of the extracted tooth was chosen to evaluate changes of horizontal and vertical bone remodeling. The baseline CBCT and bone surfaces were aligned so outlines of baseline and four months after extraction are shown over the CBCT slice mid-sagittal slice and all measurements were made on the same slice to avoid angulations measurements. The radiographic parameters measured were as follows:

- Horizontal ridge width (mm) at 2 mm apical to crest (CBCTCRW2) and 4 mm apical to crest (CBCTCRW4) at baseline and four months after extraction
- Vertical buccal/lingual cortical plate measurements (mm) assessed at one time point (four months after extraction) (Figure 3 f-h).

B) **Clinical measurements**

The clinical parameters were measured at 2 time points: after extraction and at the re-entry surgery, (Table 1) as follows:

• Keratinized tissue (KT) width at the buccal site

• Gingival thickness (GT) at 2 (GT2) and 4 mm (GT4) below the ridge crest at the buccal surface

• Horizontal ridge width at 2 and 4 mm below the crest.

**2.4.3. Impact of additional grafting on wound healing and PROs.**

Some patients required additional grafting during the implant placement procedure. The impact of this supplementary bone graft was assessed in terms of wound healing and pain levels following the procedure.

2.4.4. **Bone density**

Tactile assessment of the bone density during implant surgery was made (based on how the

tactile density of drilling the bone correlates to drilling other materials, and was used as a

clinical descriptive assessment of bone density from highest to lowest density (D1-D4) as described by Misch (Misch, 1990).

2.4.5. **Pink Esthetic Score (PES)**

The PES was assessed 6 months after crown delivery (Furhauser et al., 2005). The evaluation focused on the mesial and distal papillae, soft tissue margin and contour, alveolar process, tissue color, and texture. All measurements were conducted by a single blinded assessor to ensure consistency and objectivity.

2.4.6. **Overall patient satisfaction**

Overall patient satisfaction was assessed using a final questionnaire administered to each patient 6 months after crown delivery. Patients were asked to rate their satisfaction with the surgical treatment by responding to the question, "Are you satisfied with the provided surgical treatment?" with options for "Yes" or "No." An open-ended section was also included to allow patients to provide additional positive or negative feedback.

**2.4.7. μ-CT** **analysis of extraction socket bone cores**

Bone core samples were harvested from each patient during the implant placement and fixed in 10% neutral buffered formalin for μ-CT and histological analyses. μ-CT scanning was conducted at the University of Michigan (University of Michigan Musculoskeletal Core, Ann Arbor, MI, USA). The biopsies were processed at the School of Dentistry Pathology Laboratory.

Non-decalcified samples were scanned using a μCT system (μCT100 Scanco Medical, Switzerland) with a voxel size of 5 µm, medium resolution, 70 kVp, 114 µA, a 0.5 mm AL filter, 1000 projections/180°, and an integration time of 500 ms. The analysis utilized the manufacturer’s software, Scanco, with three different thresholds: 0 to 24.2% (0 to 242 on a grayscale of 0–1000) to segment background and soft tissue; 24.3% to 42.4% (243 to 424 on a grayscale of 0–1000) to segment bone from non-bone and 42.5% to 100% (425 to 1000 on a grayscale of 0–1000) for the segmentation of remnant particles (Figure 4 c,d,e). Parameters measured included total volume (TV), bone volume (BV), relative bone volume (BV/TV), trabecular number (Tb.N), trabecular thickness (Tb.Th), trabecular separation (Tb.Sp), apparent density (BMD), and material density (DMB). Results were displayed as mean ± (SD).

# **3 | RESULTS**

**3.3.4. Patient-Reported Pain Levels After Extraction and Implant Placement**

Pain levels were assessed using a self-report questionnaire both after extraction and implant placement. Pain was evaluated over the first week using three different scales (line, box, and verbal rating scale, Appendix B) and the intake of painkillers (Supplemental figures 6-7 and Appendix B)

For both procedures, pain levels significantly decreased over time (p<0.001) in both the test and control groups, with no significant differences between the groups. Figure 6A suggests that pain levels (line scale) were higher in the control group during the first two days following extraction, though this difference was not statistically significant. Similarly, no significant differences were found across the other scales. On day 6 after extraction, the number of painkillers used was similar between groups after Bonferroni correction (p=0.315), though it was statistically significant before correction (p=0.045 = 0.315/7). (Supplemental Figure 6B).

After implant placement, pain levels also decreased over time (p<0.001) in both groups, with no significant differences between the test and control groups. On day 5, painkiller consumption was similar between groups after Bonferroni correction (p=0.784), with a non-significant trend before correction (p=0.112 = 0.784/7). On that day, 26.7% of control patients and 64.3% of test patients were still using pain medications (Supplemental figure 7A and 7B).

### **3****.3.5. Micro-CT analysis**

Micro-CT analysis showed similar findings in new bone formation, residual particles, and connective tissue between the two groups. Relative new bone volume (BV/TV) constituted 26.08% in the test group and 26.01% in the control group. The residual particles were 13.84% and 12.25% in the control and test groups, respectively. Finally, the percentage of connective tissue was found to be 57.17% and 60.02%, respectively (Table 2 and Supplemental Table 8).

No significant differences were found in trabecular number (Tb.N), trabecular thickness (Tb.Th), or trabecular separation (Tb.Sp), with marrow thickness also comparable (Tb.Sp: test group 0.13 mm, control group 0.15 mm). Trabecular number was similar as well (test group: 7.83 mm⁻¹, control group: 7.69 mm⁻¹), suggesting comparable mineral content. Overall, micro-CT analysis revealed no significant differences between the two groups.

**3.3.6. Implant characteristics, distribution, success rate, and bone density.**

Fusion IntraLock implants, as previously described, were used in all patients. The diameters utilized were 3.3 mm, 3.8 mm, or 4.2 mm, with lengths of 9 mm, 10.5 mm, or 12 mm. All implants were placed in 3D prosthetically driven positions, with the implant size digitally planned based on CBCT and IOS measurements taken prior to the procedure. The most frequently used implant diameter was 3.8 mm, and length was 12 mm. (Supplemental Tables 6 & 7). In the test group, no failures were observed, resulting in a 100% success rate, while the control group had one failure, yielding a success rate of 93.3%; no significant differences were found between the two rates (p=1, Fisher's test). No significant differences were observed between the groups concerning bone density, with the most prevalent density being D3 for both cohorts (Supplemental Table 9).

**3.3.7.** **Impact of additional grafting on wound healing and pain after implant placement**

Patients with buccal/oral bone thickness of < 2 mm after implants were placed in the acceptable preplanned three-dimensional prosthetic position were provided additional bone grafting at the time of implant placement. In the CP group, 12 patients (80%) had buccal bone thickness under 2 mm and required grafts, while 10 (66.7%) patients in the BACM group also received bone grafts, with no statistical difference between both groups. A Fisher's exact test showed homogeneity between the groups regarding additional grafting, with a p-value of 0.682.

The differences in wound healing between the additional grafting and no-additional grafting groups were on the borderline of statistical significance (p=0.050, Mann-Whitney test). The use of additional grafting was associated with lower values of the healing index for both groups. (Supplemental Figure 11)

Regarding painkiller consumption, patients treated with additional grafting tended to use more painkillers compared to those without additional grafting treatment (p=0.075). However, the difference in consumption was more significantly influenced by the specific day of treatment (p=0.046), with the largest difference observed on days 3 and 4. This trend was similar for both the control and test groups (p=0.209). (Supplemental Figure 12)

For the verbal pain scale, additional grafting treatment resulted in a slight difference (p=0.098), with slightly higher pain assessments in the additional grafting group. This pattern was consistent across both groups (p=0.603). (Supplemental Figure 12)

**3.3.8. Pink Esthetic Score (PES) and patient satisfaction**

The PES score was higher in the test group compared to the control group; however, this difference did not reach statistical significance (p=0.202, Mann-Whitney test) (Supplemental Figure 13).

To date, 14 patients in the test group and 12 patients in the control group have been analyzed 6 months after crown delivery. All patients in both groups reported satisfaction with the surgical procedures provided.

## 3.4. **Statistical analysis**

Statistical analysis involved descriptive statistics for categorical variables (frequencies) and continuous variables (mean, SD, range, median, percentiles) were computed using SPSS software. Non-parametric methods were used for non-normally distributed data (Shapiro-Wilk test), with Mann-Whitney’s test for group comparisons and Wilcoxon’s test for intra-group comparisons (Bonferroni correction).

Ordinal pain questionnaire data were analyzed using the Brunner-Langer model for longitudinal data, assessing main effects and interactions with an ANOVA-type statistic. Chi-square and Fisher’s exact tests evaluated categorical variable homogeneity, using a significance level of 5% (α=0.05). Immunohistochemical data, analyzed with Jamovi software (Jamovi project; were described by mean and standard deviation (<https://www.jamovi.org>).

REFERENCES

Araújo, M. G., & Lindhe, J. (2009). Ridge alterations following tooth extraction with and without flap elevation: an experimental study in the dog. *Clin Oral Implants Res, 20*(6), 545-549. doi:10.1111/j.1600-0501.2008.01703.x

Chu, S. J., Sarnachiaro, G. O., Hochman, M. N., & Tarnow, D. P. (2015). Subclassification and Clinical Management of Extraction Sockets with Labial Dentoalveolar Dehiscence Defects. *Compend Contin Educ Dent, 36*(7), 516, 518-520, 522 passim.

Couso-Queiruga, E., Weber, H. A., Garaicoa-Pazmino, C., Barwacz, C., Kalleme, M., Galindo-Moreno, P., & Avila-Ortiz, G. (2023). Influence of healing time on the outcomes of alveolar ridge preservation using a collagenated bovine bone xenograft: A randomized clinical trial. *J Clin Periodontol, 50*(2), 132-146. doi:10.1111/jcpe.13744

Eskandarlou, M., Azimi, M., Rabiee, S., & Seif Rabiee, M. A. (2016). The Healing Effect of Amniotic Membrane in Burn Patients. *World J Plast Surg, 5*(1), 39-44.

Furhauser, R., Florescu, D., Benesch, T., Haas, R., Mailath, G., & Watzek, G. (2005). Evaluation of soft tissue around single-tooth implant crowns: the pink esthetic score. *Clin Oral Implants Res, 16*(6), 639-644. doi:10.1111/j.1600-0501.2005.01193.x

Hassan, M., Prakasam, S., Bain, C., Ghoneima, A., & Liu, S. S. (2017). A Randomized Split-Mouth Clinical Trial on Effectiveness of Amnion-Chorion Membranes in Alveolar Ridge Preservation: A Clinical, Radiologic, and Morphometric Study. *Int J Oral Maxillofac Implants, 32*(6), 1389-1398. doi:10.11607/jomi.5875

Holtzclaw, D. J., & Toscano, N. J. (2013). Amnion–chorion allograft barrier used for guided tissue regeneration treatment of periodontal intrabony defects: A retrospective observational report. *Clinical Advances in Periodontics, 3*(3), 131-137.

Khan, R. S., Aslam, M., Ucer, C., & Wright, S. (2023). Success of Xenografts in Alveolar Ridge Preservation Based on Histomorphometric Outcomes. *Dent J (Basel), 11*(9). doi:10.3390/dj11090215

Koob, T. J., Lim, J. J., Massee, M., Zabek, N., & Denoziere, G. (2014). Properties of dehydrated human amnion/chorion composite grafts: Implications for wound repair and soft tissue regeneration. *J Biomed Mater Res B Appl Biomater, 102*(6), 1353-1362. doi:10.1002/jbm.b.33141

Lai, V. J., Michalek, J. E., Liu, Q., & Mealey, B. L. (2020). Ridge preservation following tooth extraction using bovine xenograft compared with porcine xenograft: A randomized controlled clinical trial. *J Periodontol, 91*(3), 361-368. doi:10.1002/jper.19-0211

Lindhe, J., Cecchinato, D., Donati, M., Tomasi, C., & Liljenberg, B. (2014). Ridge preservation with the use of deproteinized bovine bone mineral. *Clin Oral Implants Res, 25*(7), 786-790. doi:10.1111/clr.12170

Löe, H. (1967). The Gingival Index, the Plaque Index and the Retention Index Systems. *J Periodontol, 38*(6), Suppl:610-616. doi:10.1902/jop.1967.38.6.610

Mermet, I., Pottier, N., Sainthillier, J. M., Malugani, C., Cairey-Remonnay, S., Maddens, S., . . . Aubin, F. (2007). Use of amniotic membrane transplantation in the treatment of venous leg ulcers. *Wound Repair Regen, 15*(4), 459-464. doi:10.1111/j.1524-475X.2007.00252.x

Monje, A., Chappuis, V., Monje, F., Munoz, F., Wang, H. L., Urban, I. A., & Buser, D. (2019). The Critical Peri-implant Buccal Bone Wall Thickness Revisited: An Experimental Study in the Beagle Dog. *Int J Oral Maxillofac Implants, 34*(6), 1328-1336. doi:10.11607/jomi.7657

Niknejad, H., Peirovi, H., Jorjani, M., Ahmadiani, A., Ghanavi, J., & Seifalian, A. M. (2008). Properties of the amniotic membrane for potential use in tissue engineering. *Eur Cell Mater, 15*, 88-99. doi:10.22203/ecm.v015a07

Nohra, J., Dagher, M., Matni, G., Mokbel, N., Jobaili, E., & Naaman, N. (2018). Effect of Primary Stability and Soft- and Hard-Tissue Thickness on Marginal Bone Loss: A Prospective Pilot Study. *Implant Dent, 27*(5), 542-546. doi:10.1097/ID.0000000000000810

O'Leary, T. J., Drake, R. B., & Naylor, J. E. (1972). The plaque control record. *J Periodontol, 43*(1), 38. doi:10.1902/jop.1972.43.1.38

Saleh, M. H. A., Couso-Queiruga, E., Ravida, A., Dukka, H., Paiva De Andrade, N., Ou, A., & Wang, H. L. (2022). Impact of the periodontal phenotype in premolar and molar sites on bone loss following full-thickness mucoperiosteal flap: A 1-year prospective clinical trial. *J Periodontol, 93*(7), 966-976. doi:10.1002/JPER.21-0591

Saleh, M. H. A., Dias, D. R., & Kumar, P. (2024). The economic and societal impact of periodontal and peri-implant diseases. *Periodontol 2000*. doi:10.1111/prd.12568

Sikkerimath, B. C., Dandagi, S., Gudi, S. S., & Jayapalan, D. (2012). Comparison of vestibular sulcus depth in vestibuloplasty using standard Clark's technique with and without amnion as graft material. *Ann Maxillofac Surg, 2*(1), 30-35. doi:10.4103/2231-0746.95313

Silness, J., & Loe, H. (1964). Periodontal Disease in Pregnancy. Ii. Correlation between Oral Hygiene and Periodontal Condtion. *Acta Odontol Scand, 22*, 121-135. doi:10.3109/00016356408993968

Spray, J. R., Black, C. G., Morris, H. F., & Ochi, S. (2000). The influence of bone thickness on facial marginal bone response: stage 1 placement through stage 2 uncovering. *Ann Periodontol, 5*(1), 119-128. doi:10.1902/annals.2000.5.1.119

Velez, I., Parker, W. B., Siegel, M. A., & Hernandez, M. (2010). Cryopreserved amniotic membrane for modulation of periodontal soft tissue healing: a pilot study. *Journal of periodontology, 81*(12), 1797-1804.

Wang, H.-L., & Tsao, Y.-P. (2007). Mineralized bone allograft-plug socket augmentation: rationale and technique. *Implant dentistry, 16*(1), 33-41.
