# Supplementary material for: The effect of bioactive membrane in alveolar ridge preservation: A double‐blind randomized clinical trial
Source: J Periodontol. 2026 Feb 26;97(9):1871–84. doi: 10.1002/jper.70094 (PMC13568902; doi:10.1002/jper.70094)
Supplement: Supplementary file 3 [file JPER-97-1871-s003.docx]

Supplemental Figure 1

Supplemental Figure 2

Supplemental Figure 3

Supplemental Figure 4

A) Changes in Wound Healing Index by Group

%

|  | 2 | 3 | 4 | 5 |
| --- | --- | --- | --- | --- |
| CONTROL T2w | 13.3 | 46.7 | 20 | 20 |
| CONTROL T4w | 6.7 | 20 | 46.7 | 26.7 |
| CONTROL T12w |  |  | 6.7 | 93.3 |
| TEST T2w |  | 53.3 | 40 | 6.7 |
| TEST T4w |  | 13.3 | 40 | 46.7 |
| TEST T12w |  |  |  | 100 |

B) Wound Healing Index at 2w after IP by Group

%

|  | 2 | 3 | 4 | 5 |
| --- | --- | --- | --- | --- |
| CONTROL | 13.3 | 26.7 | 13.3 | 46.7 |
| TEST | 6.7 | 13.3 | 13.3 | 66.7 |

Supplemental Figure 5.

| **Supplemental figure 6A**. Pain: Verbal scale Day 1 to 7 after ARP by group   | **Supplemental figure 6B**. Number of painkillers per group after ARP   |
| --- | --- |
|  |  |

| **Supplemental figure 7A**. Pain assessment/ line scale   | **Supplemental figure 7B**. Relative effect plot of numbers of painkiller after IP per group   |
| --- | --- |

Supplemental Figure 8

Supplemental Figure 9

Wound Healing Index at 2w after IP by GBR

%

|  | 2 | 3 | 4 | 5 |
| --- | --- | --- | --- | --- |
| Non-GBR |  |  | 12.5 | 87.5 |
| GBR | 13.6 | 27.3 | 13.6 | 45.5 |

| (a) Painkillers taken. Comparison between additional grafting vs non additional grafting patients |   (b) Patient reported verbal scale of pain. Comparison between additional grafting vs non additional grafting patients |   (c)Patient reported verbal scale of pain. Comparison between additional grafting vs non additional grafting in control and test groups. |
| --- | --- | --- |

*Supplemental Figure 10*

*Supplemental Figure 11*
